# Developing predictive models for COVID-19 positive tests based on the XGBoost and random forest algorithms with internet search data

**DOI:** 10.1186/s12889-025-25569-w

**Published:** 2025-11-28

**Authors:** Yikun Chang, Jinwei Chen, Xiaoxuan Chen, Yueqian Wu, Hui Tang, Gonghua Wu, Jie Sun, Yuyi Liao, Haolin Chen, Senyao Cai, Yuantao Hao, Wangjian Zhang, Zhicheng Du

**Affiliations:** 1 Department of Medical Statistics, School of Public Health & Sun Yat-sen Global Health Institute & Center for Health Information Research, Sun Yat- sen University, Guangzhou, China; 2 Peking University Center for Public Health and Epidemic Preparedness & Response, Beijing, 100191 China; 3 Department of Epidemiology & Biostatistics, School of Public Health, Peking University, Beijing, 100191 China; 4 Key Laboratory of Epidemiology of Major Diseases (Peking University), Ministry of Education, Beijing, 100191 China; 5 Guangzhou Joint Research Center for Disease Surveillance and Risk Assessment, Sun Yat-sen University & Guangzhou Center for Disease Control and Prevention, Guangzhou, China

**Keywords:** Baidu search index, Time-lagged correlation analysis, Machine learning, Feature selection

## Abstract

**Background:**

Although strategies for COVID-19 have shifted towards normalized measures globally, establishing predictive models based on Internet search data remains crucial for swiftly controlling and preventing future outbreaks. This study aims to utilize Internet search data for early epidemic surveillance and warning.

**Methods:**

We collected the daily number of COVID-19 positive tests and the daily Baidu Search Index (BSI) of COVID-19 related keywords. First, we screened keywords with a maximum correlation coefficient exceeding 0.9 by time-lagged correlation analysis. Then, we used the original and lagged BSI to construct XGBoost and Random Forest (RF) models for short-term prediction of the COVID-19, respectively. Next, we selected top 5 important predictors according to the importance gain in XGBoost model and constructed a comprehensive search index (CSI) weighted by the importance gain. Finally, we used the distributed lagged nonlinear model (DLNM) to evaluate the relationship between the CSI and the number of COVID-19 positive tests.

**Results:**

We identified 20 keywords had a maximum correlation coefficient exceeding 0.9 with lag days of 1–10 days. Then, we found that the predictive performance of the XGBoost models was better than that of the RF models. And XGBoost models using lagged BSI (compared to original BSI) had a better predictive performance for forecasting 3 days, with an RMSE of 803.85 and a MAPE of 9.96%. Finally, we observed that the CSI was statistically associated with the number of COVID-19 positive tests, with the maximum relative risks (RR) at lags of 0, 3, 5, and 7 days being 2.18 (95%*CI* 1.60–2.97), 1.94 (95%*CI* 1.10–3.43), 1.86 (95%*CI* 1.01–3.44), and 2.03 (95%*CI* 1.00-4.11), respectively.

**Conclusions:**

The XGBoost model with the lagged BSI can predict COVID-19 epidemics, which make it a powerful addition to the traditional surveillance systems.

**Supplementary Information:**

The online version contains supplementary material available at 10.1186/s12889-025-25569-w.

## Introduction

The COVID-19 pandemic was a public health emergency of international concern that profoundly affected the whole world, imposing a severe health and social burden on all countries. In the coming years, it is possible for COVID-19 to evolve into a seasonal or persistent epidemic like influenza. Therefore, establishing timely surveillance and early warning systems for COVID-19 is of paramount importance. In the current context, most countries have shifted from active screening strategies to traditional passive surveillance for COVID-19. This approach relies mainly on data such as confirmed cases and laboratory diagnostic indicators to monitor and provide warnings about future outbreak trends [1–3]. Unfortunately, these data often lag behind actual outbreak trends [4], and it is difficult to accurately track the development of the epidemic. Hence, achieving early surveillance and timely warning has become an urgent issue.

In the era of big data, there is a growing focus on using Internet search data as a novel source for disease surveillance. The Internet plays a key role in health information searches [5]. The public increasingly turns to the Internet for information about epidemics, treatment methods, and medication purchases. Monitoring health-related Internet search data can reveal the prevalence of specific diseases. Internet search data have proven effective in detecting the initial signs of an epidemic [6]. Compared to traditional data, Internet search data can predict disease outbreaks days or even weeks in advance [7, 8]. Therefore, the use of Internet search data as a complement to traditional disease surveillance indicators could lead to improved early monitoring and warning of epidemics.

Over the past two decades, widely utilized Internet search engines like Google Trends and Baidu Index have served as monitoring platforms for various diseases, including influenza [9], malaria [10], tuberculosis [11], hand, foot, and mouth disease [12], avian influenza [13], and dengue fever [14], etc. Indeed, during the COVID-19 pandemic, Internet search data were also successfully used for surveillance and early warning of COVID-19 [15–17]. For example, a study [18] used weather factors and Google data to fit an ARIMA model to predict the spread of COVID-19. A Random Forest (RF) model was used in conjunction with Google Trends to predict the incidence of COVID-19 in 215 countries and territories [19]. Additionally, researchers used data mining and deep learning techniques to model Google Trends data to predict the incidence of COVID-19 in Iran [20]. There have also been studies using Google Trends and RF algorithms to accurately predict the alert level of COVID-19 in 202 countries [21]. Consequently, developing predictive models using Internet search data stands out as a promising approach for achieving early surveillance and timely warning of COVID-19.

Since Google Trends is not available in China, Baidu, as the most widely used search engine in the country [22], serves as a valuable alternative for capturing public health-related search data. For example, Using time-lagged cross-correlation analysis, Gong et al. [23] found that the Baidu Index for the keywords “Novel Coronavirus”, “pneumonia”, “Novel pneumonia”, “Novel Coronavirus pneumonia”, “epidemic”, “Wuhan” and “Wuhan pneumonia” were significantly correlated with the case data in China, with the presence of a 0–4 day advance. Some researchers also correlated the Baidu index of four categories of keywords such as prevention, symptoms, treatment, and terminology in COVID-19 subject terms with the incidence data in China and optimized the Least Squares Support Vector Machine (LSSVM) model using three intelligent optimization algorithms (Multiverse optimizer, Slime mould algorithm and Equilibrium optimizer) [24]. However, the current research on the application of Baidu Search Index to COVID-19 prevalence trends in China has several limitations. First, there is a lack of research investigating the relationship between Baidu Search Index keywords and COVID-19 prevalence following the complete removal of control measures. Furthermore, previous studies frequently lack a comprehensive search strategy, potentially ignoring some relevant keywords such as symptoms and treatment related to COVID-19. Second, few studies have adequately considered the lag effects of different keywords, limiting the ability to capture the complex dynamics of epidemic trends. Third, previous studies have often focused on the relationship between individual keywords and COVID-19 trends. However, real-world decisions cannot rely on a single indicator alone, which limits the effectiveness of decision-making. How to build efficient predictive models and identify effective predictors after the lifting of COVID-19 restrictions is crucial and remains an urgent issue.

Therefore, this study makes several important contributions. First, it is the first to explore the relationship between the Baidu Search Index and COVID-19 outbreaks after the lifting of restrictions, thereby filling a critical research gap. We constructed a comprehensive directory of COVID-19-related keywords using a comprehensive search strategy and further optimized the machine learning predictive model through lag processing to account for the delayed effects of different keywords. This approach not only provides valuable references for public health strategies during this phase but also offers a complete theoretical framework for future research. Second, by identifying and combining key potential search keywords into a comprehensive search index, we underscore the importance of utilizing multiple keywords to improve outbreak monitoring. These contributions not only advance real-time surveillance but also provide actionable insights for public health authorities to implement more targeted and timely interventions.

## Methods

**Fig. 1:**
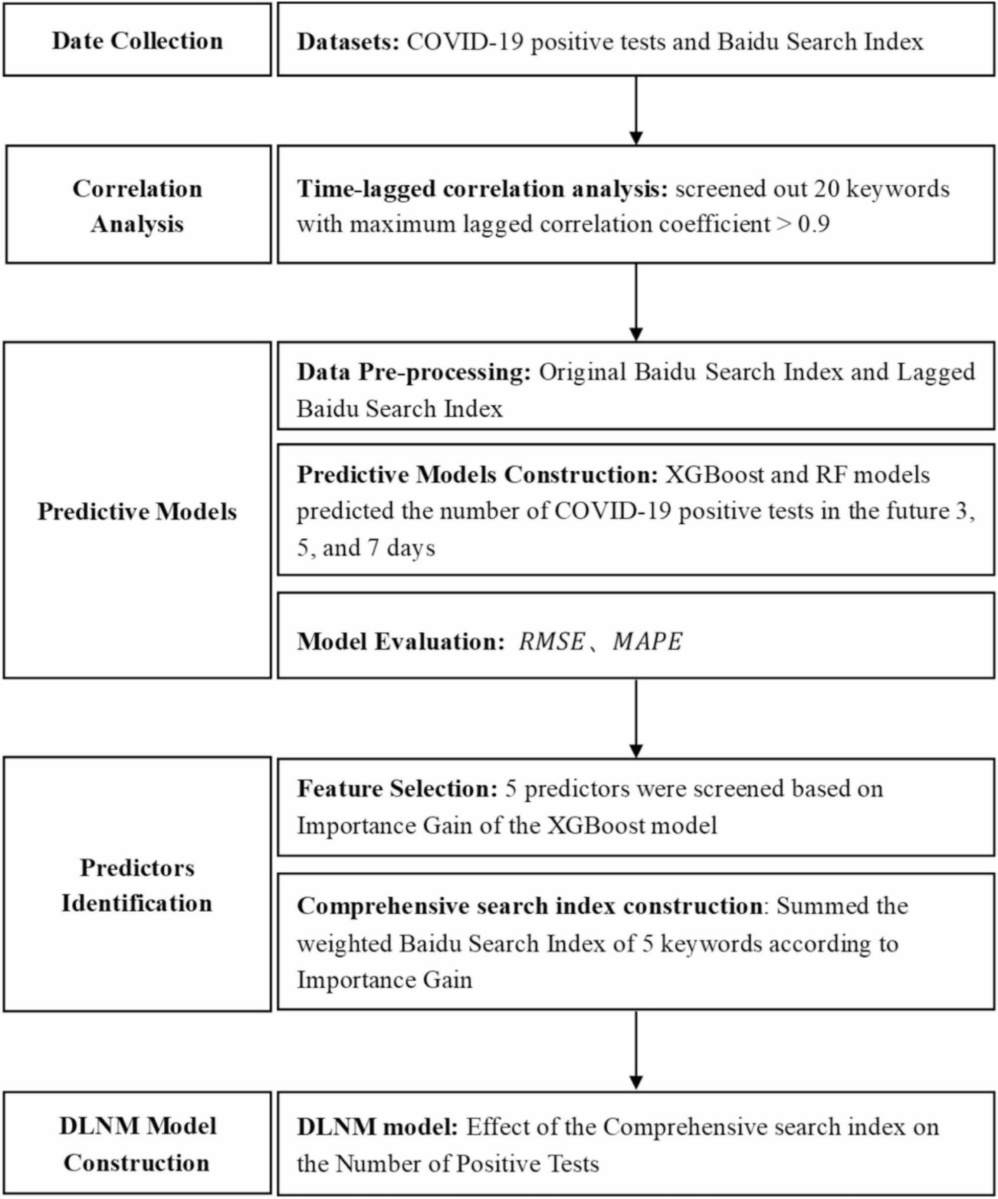
Technology roadmap

The overall technology roadmap of this study is shown in Fig. 1.

### Data collection

#### COVID-19 positive tests data

During a sudden COVID-19 outbreak, traditional confirmed cases may not accurately reflect the actual trend of the epidemic, while the test data can be used to forecast the actual scale of infection [25]. Therefore, the first dataset used in this study was the daily number of COVID-19 positive tests in China. The Chinese Center for Disease Control and Prevention (CDC) published the nationwide results of novel coronavirus nucleic acid testing from November 1, 2022, to April 27, 2023. We extracted the number of COVID-19 positive tests dataset using Engauge-Digitizer to construct the daily time series of COVID-19 positive tests.

#### Baidu search index

**Fig. 2:**
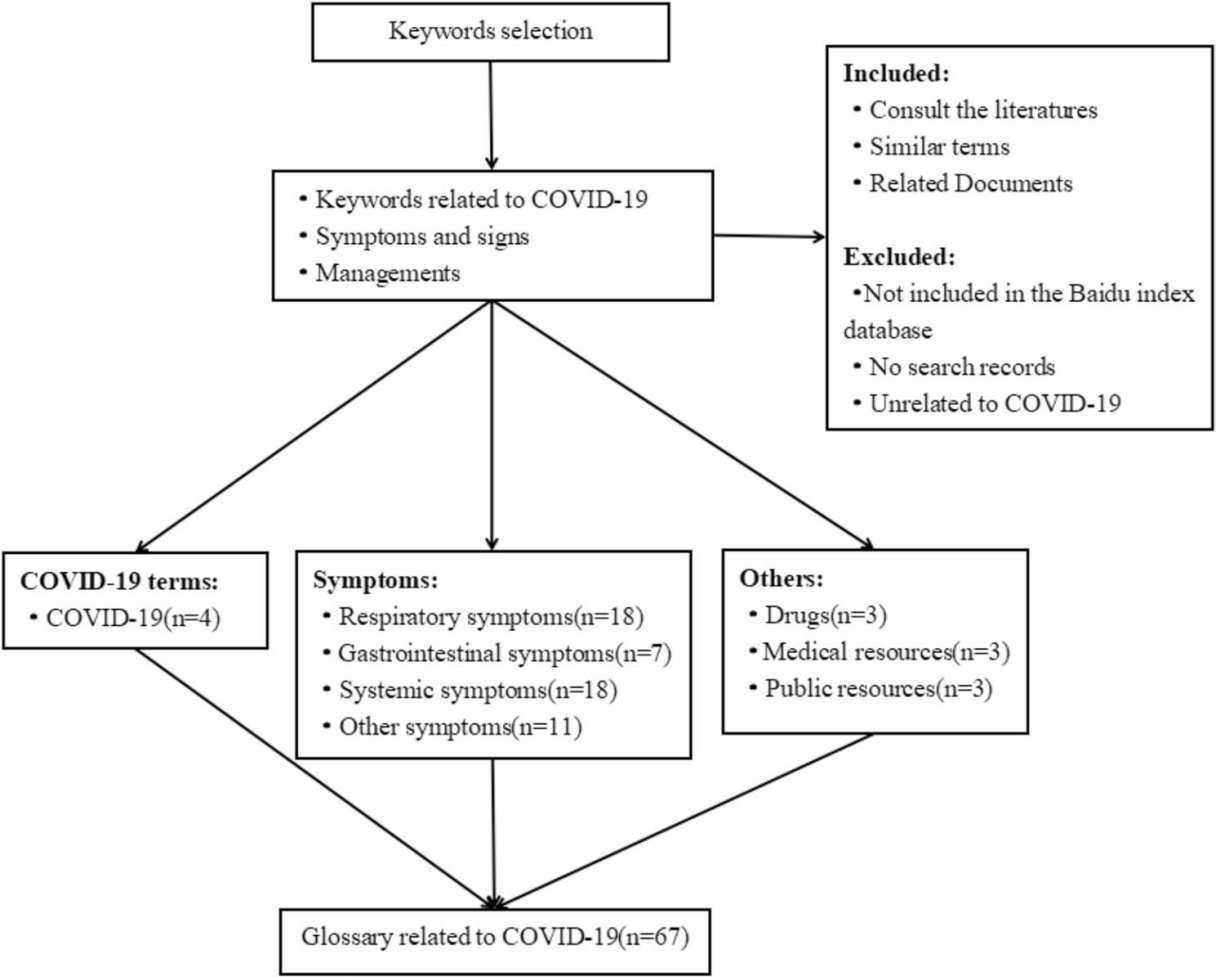
COVID-19-related keywords selection procedure

The selection of COVID-19-related keywords is a key aspect in the establishment of prediction models, which can help us identify potential predictors. At present, there are three main methods of keyword selection [26] in common use: Technical access method, which involves using computing devices to incorporate all possible keywords into the study; Scope access method, where a scope of keywords is identified and then selected within that scope; Direct access method, which involves using subjective experience to select keywords. We employed a combination of “scope access method” and “direct access method” strategies to construct a directory of Internet search keywords related to COVID-19. The construction process is shown in Fig. 2, we first searched COVID-19-related literature from PubMed, Web of Science, and other major databases, and used the scope access method to categorize COVID-19-related keywords into three broad scopes, including the COVID-19 terms, signs and symptoms, and managements. For the signs and symptoms of COVID-19, we selected a more precise scope based on the manifestations of different systems throughout the body. Finally, based on practical experience and retrieved literature [27], we selected initial keywords within the three identified scopes using the direct access method. After expert consultation, we identified a total of 67 keywords in 6 categories to construct a glossary related to COVID-19 in Supplementary Table S1.

Baidu is the most common search engine in China. In June 2024, Baidu App monthly active users reached 703 million [22]. Moreover, Baidu Index is easily accessible and provides real-time data. Therefore, the trend of Baidu Search Index can predict COVID-19 trends in China to a certain extent. Baidu Search Index (BSI) is based on the search volume, scientifically analyzing, and calculating the weighted sum of search frequency of each keyword in Baidu engine. The larger the BSI, the more people are searching. We used Baidu Index platform to search for keywords, and extracted daily BSI (including data on mobile and PC terminals) of the selected keywords from November 1, 2022, to April 27, 2023, through Python’s qdata module.

### Statistical description

General statistical description of the daily number of COVID-19 positive tests in China and the BSI of each keyword were carried out. We calculated the maximum and minimum values and the peak date of the BSI for each keyword and the number of positive tests. Temporal trends were showed for the BSI time series of different keywords by plotting the temporal trend.

### Correlation analysis

Time-lagged correlation analysis can be used to calculate the correlation between two time series variables at any different moments. Since we are more interested in using the BSI at the current moment to predict the number of positive tests at a future moment, in this study, we defined lag days as the number of days ahead of the BSI for each keyword relative to the number of COVID-19 positive tests. For example, a lag of 1 day means that the number of positive tests corresponds to the BSI for the keyword from the previous day. We calculated the lagged correlation between the BSI for each keyword with a lag day of 1–14 days and the number of daily COVID-19 positive tests at the current moment. Through time-lagged correlation analysis, we obtained the maximum correlation coefficient between the BSI and the number of COVID-19 positive tests for each keyword, as well as the lag days corresponding to reaching the maximum correlation coefficient. Finally, we screened keywords with a maximum lagged correlation coefficient > 0.9 for the next step in model construction.

### Predictive models

#### Data Pre-processing

The BSI of the keywords identified through time-lagged correlation analysis will be used as predictors to construct RF and XGBoost predictive models. We constructed two time series datasets: the original Baidu Search Index (original BSI) dataset and the lagged Baidu Search Index (lagged BSI) dataset of each keyword, respectively. The original BSI dataset remains unprocessed. The lagged BSI dataset is based on the maximum lag correlation coefficient to determine the number of lag days for each keyword, and then the corresponding BSI data is shifted backward by the corresponding number of lag days to reflect its lagged effect in time. Then, we divided the BSI datasets and the COVID-19 positive tests dataset into the training sets for fitting the model, and the testing sets for verifying the model performance separately. Since the 7-day window [19] provides more information for decision-making and aids competent authorities in timely intervention, the testing sets were the last 3, 5, and 7 days of datasets respectively, and the rest of the data was taken as the training sets.

#### Predictive models construction

Machine learning models have proven effective in many applications. Both the XGBoost model and the RF model excel in handling situations that do not depend on the distribution of data and involve complex nonlinear relationships. Additionally, they are also effective in handling high-dimensional data and performing variable selection. Therefore, these two models were used in this study to construct predictive models for short-term prediction for the future number of COVID-19 positive tests while also identifying potential predictive factors. The original BSI dataset and the lagged BSI dataset were used as feature variables for the models, respectively, and the number of positive tests dataset was used as the target variable. For each prediction day (3, 5, and 7 days), we constructed the XGBoost models and RF models using the original BSI and lagged BSI dataset, respectively. Then, these models were trained using the training set, and hyperparameter tuning was performed using 5-fold cross-validation to improve the performance of the model. Finally, we used the fitted models to predict the number of test positives for the next 3, 5, and 7 days on the testing sets and compared them with the actual data. For comparison, we also constructed traditional baseline models, including ARIMA and ARIMAX, to evaluate the added value of machine learning approaches.

#### XGBoost model

The XGBoost model is a powerful ensemble learning method based on decision trees. It employs second-order Taylor expansion [28] to approximate the loss function, while incorporating regularization terms into the objective function to effectively prevent overfitting problems [29]. It provides excellent predictions by converting a set of weak learners into a strong learner. The objective function of the XGBoost model is as follows:

$$\:{Y}^{k}=\sum\:_{i=1}^{n}l\left(\left({y}_{i},{y}_{i}^{k-1}\right)+{f}_{k}\left({x}_{i}\right)\right)+\varOmega\:\left({f}_{k}\right)$$

where $\:n$ represents the sample size, 178 using original BSI dataset and 168 using lagged BSI dataset, respectively, *k* represents the number of iterations, $\:{x}_{i}$ and $\:{y}_{i}$ are the Baidu Search Index of different keywords and the corresponding number of COVID-19 positive tests at the $\:{i}^{t}$ sample, respectively. $\:l$ is a loss function that calculates the difference between the number of COVID-19 positive tests and the final prediction plus the new tree output. $\:{y}_{i}^{k-1}$ is the prediction of the $\:{i}^{t}$ sample at the $\:{k-1}^{t}$ iteration, $\:{f}_{k}\left({x}_{i}\right)$ is the prediction of the $\:{i}^{t}$ sample by the current learner, and $\:\varOmega\:\left({f}_{k}\right)$ is the regularization term of the $\:{k}^{t}$ learner.

#### Random Forest model

Random Forest (RF) model is an ensemble learning algorithm [30]. The original data set is randomly sampled to form n distinct sample data sets, from which n decision tree models are constructed. Finally, it makes predictions according to the average value of these decision tree models. The objective function of the RF model is as follows:

$$\:Y=\:\frac{1}{N}\sum\:_{i=1}^{N}{f}_{i}\left(x\right)$$

where $\:x$ is the BSI of different keywords, $\:N$ is the number of trees, $\:i$ represents the $\:{i}^{t}$ tree, $\:{f}_{i}\left(x\right)$ donates the $\:{i}^{t}$ tree predicts the number of positives based on the input $\:x$. $\:Y$ is the number of positive tests predicted by the RF model.

#### Models Evaluation

The performance of the model was evaluated using the testing sets after the model fitting was completed and the model predictions were compared to the actual number of positive tests. We compared the predictive performance of different models as well as different input feature variables to select the best predictive model. Root mean squared error (RMSE) and mean absolute percentage error (MAPE) were used to evaluate the model performance. These matrices used the following formulas:

$$\:RMSE=\sqrt{\frac{1}{n}\sum\:_{i=1}^{n}{\left({\widehat{y}}_{i}-{y}_{i}\right)}^{2}}$$

$$\:MAPE=\frac{100\%}{n}\sum\:_{i=1}^{n}\left|\frac{{\widehat{y}}_{i}-{y}_{i}}{{y}_{i}}\right|$$

where $\:{y}_{i}$ and $\:{\widehat{y}}_{i}$ are the actual number of COVID-19 positive tests and model predicted number of COVID-19 positive tests respectively and $\:n$ denotes the number of observations. When RMSE and MAPE are close to 0, the model is more efficient.

### Predictors identification

#### Feature selection

As the prediction range increased from 3 days to 5 days and then to 7 days, the error rate of the model increased. After comparing the performance of the models, we selected the best one and utilized the feature selection function of the XGBoost model to identify significant predictors based on the importance gain. We added a random sequence to the optimal model, and then reconstructed the model again based on the parameters of the optimal model to obtain the importance gain of the BSI of each keyword. Higher importance gain represents higher significance [31]. Feature importance analysis aims to identify key predictive variables. We empirically selected features with importance gains greater than 0.01 [32]. The 5 keywords were indentified as the most important contributors under this criterion.

#### Comprehensive search index construction

We identified five key search keywords that directly reflect public concerns about health issues. However, the relationship between different keywords and the number of COVID-19 positive tests varies at different stages of the epidemic. Due to the influence of multiple keywords, relying on a single keyword’s search trend for decision-making is inadequate. Therefore, we developed a comprehensive search index (CSI) to intuitively reflect epidemic trends and facilitate better decision-making. We obtained the importance gain of the variables based on feature selection. Then, we used the importance gain of the variables as weights, weighted and summed the BSI of the screened keywords to construct a CSI to investigate their relationship with the number of COVID-19 positive tests. This approach aims to enhance the effectiveness of monitoring and early warning by focusing on one or a few key keywords, while also identifying critical factors in epidemic prediction to provide a scientific basis for public health decision-making, thereby facilitating practical application and dissemination.

### DLNM model

To explore the effect of the CSI on the number of COVID-19 positive tests and verify the effectiveness of the selected predictors, we constructed the distributed lagged nonlinear model (DLNM). The DLNM model can study the nonlinear relationship between one or multiple variables and the response variables by constructing a cross-basis [33], offering flexibility in studying the associations involving nonlinearities and lag effects in time series data. The objective function of DLNM model is shown as follows:

$$\:g\left({\mu\:}_{t}\right)=\alpha\:+\sum\:_{j=1}^{J}{f}_{j}\left({x}_{tj};{\beta\:}_{j}\right)+\sum\:_{k=1}^{K}{\gamma\:}_{k}{\mu\:}_{tk}$$

where $\:{\mu\:}_{t}\equiv\:E\left({Y}_{t}\right)$, $\:g$ is a monotonic link function, here we use the quasi-Poisson distribution, *t* represents time, $\:{Y}_{t}$ is the number of COVID-19 positive tests, $\:{x}_{t}$ is the comprehensive search index, $\:{\mu\:}_{tk}$ is the linear effect of other confounding factors, $\:{\beta\:}_{j}$, $\:{\gamma\:}_{k}$ is the corresponding coefficient, $\:{f}_{j}$ is the cross-basis function of various explanatory variables, which is to respectively select the appropriate basis function for the relationship between independent variable and response variable, the distribution of hysteresis effect.

### Statistical tools

All statistical analysis methods were performed using R (version 4.3.1). The “ccf” function in R was applied to explore the time-lagged correlation between the BSI of COVID-19-related keywords and the daily number of COVID-19 positive tests in China during the period from November 1, 2022 to April 27, 2023. The “cor” function in R calculated Pearson correlation coefficient between different keywords. The package “xgboost” and “randomForest” in R were used to construct XGBoost model and RF model, and the package “dlnm” was used to build DLNM model. $\:P<.05\:$was considered statistically significant.

## Results

### Statistical description

Table 1 shows the general characteristics of the daily number of COVID-19 positive tests and the BSI for each keyword. The number of positive tests during the study period ranged from a minimum of 409 to a maximum of 6,950,750, with the peak date occurring on December 22, 2022. At the same time, it was observed that the peak date of the BSI for the 2 keywords “Rigor (Han Chan)” and “Rash” coincided with the date of the highest number of COVID-19 positive tests, which was December 22, 2022 for both. In addition, the BSI for 37 keywords reached its peak search volume before the peak date of the number of positive tests, ranging from 1 to 44 days earlier.

**Table 1:** Statistical description of the daily number of COVID-19 positive tests and BSI for different keywords

No.	Variable (in Chinese)^a^	Min	Max	Median	Mean	SD^b^	Peak date^c^
1	number of positive tests	409	6,950,750	8338	494678.725	1340810.901	2022/12/22
2	Novel Coronavirus Pneumonia	199	8528	381	1866.742	2338.694	2022/11/27
3	Novel Coronavirus Infection	69	2070	224.5	282.242	213.336	2022/12/27
4	COVID-19(Xin Guan Fei Yan)	1016	27,271	2356.5	5882.253	6423.264	2022/12/24
5	COVID-19(Xin Guan Gan Ran)	0	7528	313	533.820	975.728	2022/12/27
6	Cough	2226	35,902	5066	7262.972	7125.186	2022/12/21
7	Dry Cough	622	7399	1041.5	1603.534	1499.336	2022/12/21
8	Expectoration	122	1395	278	401.556	304.360	2022/12/23
9	Rhinorrhea (Liu Ti)	69	326	141.5	142.427	55.859	2022/12/18
10	Rhinorrhea (Liu Bi Ti)	189	2164	655.5	722.140	397.850	2022/12/20
11	Rhinobyon	576	15,804	1097	2140.831	3224.753	2022/12/23
12	Runny nose (Pen Ti)	443	1231	1013	980.449	142.617	2022/12/19
13	Runny nose (Da Pen Ti)	997	2706	1359	1447.489	366.622	2022/12/25
14	Pharyngalgia	152	2202	399.5	531.601	458.822	2022/12/21
15	Hoarseness	148	704	271	280.298	64.184	2023/1/6
16	Acute laryngitis	312	4442	965.5	1064.494	706.579	2022/12/26
17	Bronchitis	2388	13,111	3999.5	4908.039	2490.661	2022/12/29
18	Wheeze	317	582	455	458.208	45.346	2022/12/27
19	Asthma	1491	4200	2464.5	2553.601	471.782	2022/12/26
20	Sore throat	934	11,560	1499.5	2136.685	2279.226	2022/12/21
21	Hypopnea	99	380	200	204.573	45.229	2022/12/26
22	Dyspnea	425	1423	635	705.146	196.961	2022/12/27
23	Shortness of breath	473	2483	616	742.287	344.468	2022/12/27
24	Chest tightness	998	4137	1157.5	1404.140	616.927	2022/12/28
25	Chest pain	828	1417	1015.5	1024.843	69.087	2022/12/29
26	Palpitate (Xin Huang)	934	1815	1055	1107.702	183.759	2022/12/27
27	Palpitate (Xin Ji)	3268	14,877	4472.5	5208.146	2162.066	2022/12/26
28	Fever (Fa Re)	299	13,910	755.5	1004.590	1209.656	2022/12/16
29	Fever (Fa Shao)	1126	80,211	3015	6928.579	12286.197	2022/12/17
30	Rigor (Han Chan)	419	1113	548.5	591.213	150.583	2022/12/22
31	Rigor (Han Zhan)	839	8967	1226	1367.511	654.173	2023/1/9
32	General malaise	73	619	159.5	178.107	100.336	2022/12/21
33	Fatigue (Fa Li)	192	1148	390	458.084	206.685	2022/12/20
34	Fatigue (Pi Bei)	229	963	544.5	578.500	154.371	2023/4/17
35	Myalgias	289	2328	497.5	609.270	390.092	2022/12/16
36	Backache	521	1060	865.5	858.691	115.646	2022/12/19
37	Arthralgia	148	500	264.5	272.961	54.294	2022/12/21
38	Headache (Tou Tong)	809	4118	1128	1327.163	628.871	2022/12/21
39	Headache (Tou Teng)	1142	12,370	2233	3033.388	2265.999	2022/12/20
40	Vertigo	564	1148	1055.5	1040.713	83.002	2023/3/20
41	Dizziness	1043	5556	2026.5	2206.393	832.485	2022/12/20
42	Abdominal pain (Fu Tong)	349	687	547.5	534.472	66.012	2022/11/8
43	Abdominal pain (Du Zi Tong)	569	943	828	821.961	72.443	2022/11/19
44	Abdominal pain (Du Zi Teng)	1164	4522	1571.5	1613.326	295.455	2023/1/19
45	Diarrhea (Fu Xie)	988	5251	1237.5	1406.056	514.088	2023/1/2
46	Diarrhea (La Du Zi)	1806	10,890	3019.5	3185.489	1146.362	2023/1/2
47	Nausea	798	1893	1023.5	1102.118	194.315	2022/11/26
48	Vomiting	888	1594	1015.5	1055.112	121.520	2022/12/20
49	Rash	945	1805	1110	1141.028	141.017	2022/12/22
50	Conjunctivitis	2114	20,429	3397.5	3787.657	1722.995	2023/4/18
51	Conjunctival congestion	151	320	265	262.607	27.620	2023/4/21
52	Eyeball congestion	110	238	194	185.736	31.579	2022/12/19
53	Red eyes	227	464	322	325.837	39.635	2022/12/18
54	Itchy eyes	552	1045	711	760.169	135.422	2023/4/3
55	Eyes hurt (Yan Jing Teng)	539	1228	777	803.213	131.334	2022/12/19
56	Eyes hurt (Yan Jing Tong)	215	726	338	360.191	87.706	2022/12/19
57	Smell	0	2070	396.5	451.517	455.527	2022/12/26
58	Taste	164	1801	263	395.669	345.132	2022/12/21
59	Epilepsy	2657	21,648	4615.5	4770.983	1633.581	2022/11/23
60	Lianhuaqingwen	805	83,877	1921	9371.461	17755.201	2022/12/12
61	Lianhuaqingwen capsule	1169	162,797	3680.5	18934.663	37619.683	2022/12/14
62	Isolation	373	1993	661.5	864.292	384.787	2022/11/28
63	COVID-19 vaccine	572	37,440	1158	3758.691	5394.702	2022/11/29
64	Ibuprofen	5661	420,289	11177.5	43047.270	91900.375	2022/12/16
65	Nasopharyngeal swab	97	1164	251	444.112	339.759	2022/11/29
66	COVID-19 antigen	0	6866	218.5	645.129	1361.695	2022/12/15
67	Nucleic Acids Detection	721	27,788	1724	6417.247	7890.372	2022/12/4
68	Mask	896	11,085	1223.5	2201.618	1961.156	2022/12/16

^a^When different keywords are the same in English, the keywords are displayed in English (Chinese pinyin) format

^b^SD: Standard Error

^c^Peak date: Date when the maximum value was reached

**Fig. 3:**
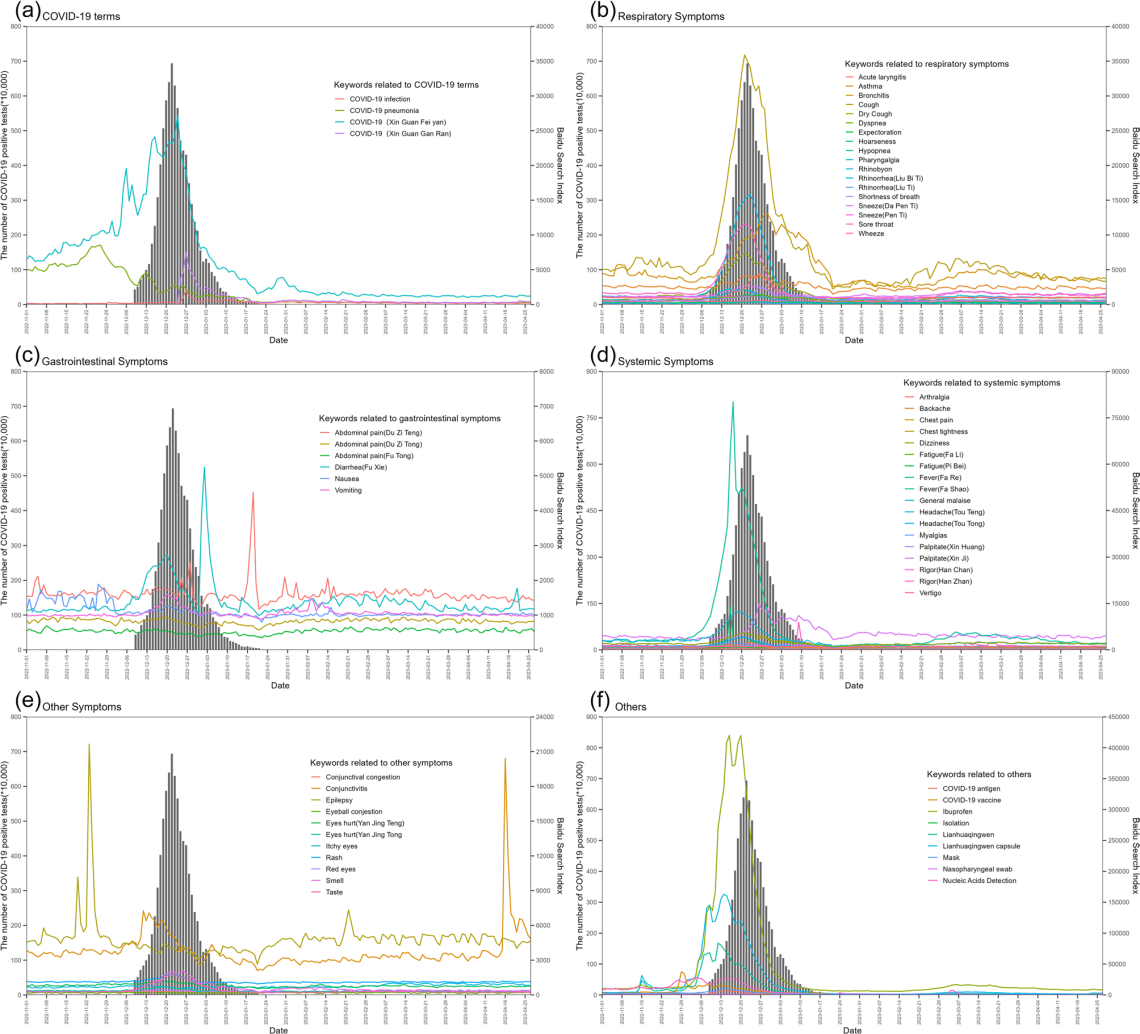
Time trend chart of BSI for different keywords and the number of COVID-19 positive tests **a** Keywords related to COVID-19 terms; **b** Keywords related to respiratory symptoms; **c** Keywords related to gastrointestinal symptoms; **d** Keywords related to systemic symptoms; **e** Keywords related to other symptoms; **f** Keywords related to others. (X axis: date, Left Y axis: the number of COVID-19 positive tests (*10,000), Right Y axis: Baidu Search Index (BSI)

The temporal trends of BSI for various keywords and the number of COVID-19 positive tests are shown in Fig. 3. Since December 9, 2022, the number of positive tests has exhibited a fluctuating trend, peaking at 6.94 million on December 22, 2022, followed by a downward trend. Among the 67 keywords collected in six categories, the top three most searched keywords in each category were the following: “COVID-19 (Xin Guan Fei Yan)”, “Novel Coronavirus Pneumonia”, “COVID-19 (Xin Guan Gan Ran)”, “Cough”, “Rhinobyon”, “Bronchitis”, “Fever (Fa Shao)”, “Palpitate (Xin Ji)”, “Fever (Fa Re)”, “Diarrhea (La Du Zi)”, “Diarrhea(Fu Xie)”, “Abdominal pain(Du Zi Teng)”, “Epilepsy”, “Conjunctivitis”, “Smell”, “Ibuprofen”, “Lianhuaqingwen”, and “Lianhuaqingwen capsule”, respectively. It is important to mention that there were significant fluctuations over time in the BSI for most of the keywords during the study period were observed, and the changes approximately corresponded with the temporal trend observed in the number of positive tests. Specifically, the temporal trends of the keywords related to COVID-19 terms (Fig. 3(a) were more consistent with the number of positive tests, and both the upward and downward trends preceded the temporal trend of the number of COVID-19 positive tests. Likewise, similar temporal patterns were observed for “Cough” in the keywords related to Respiratory symptoms (Fig. 3(b), “Vomiting” in the keywords related to Gastrointestinal symptoms (Fig. 3(c), “Fever (Fa Shao)”, “Fever (Fa Re)”, and “Headache (Tou Teng)” in the keywords related to Systemic symptoms (Fig. 3(d), “Epilepsy” in the keywords related to Other symptoms (Fig. 3(e), and “Ibuprofen”, “Lianhuaqingwen”, and “Lianhuaqingwen capsule” in the keywords related to Others (Fig. 3(f). By the end of 2022, the BSI for COVID-19-related keywords increased dramatically, especially for “Ibuprofen” with the BSI increasing nearly 27 times from 15,000 at the beginning of December 4, 2022, to a peak of about 420,000 on December 16.

### Correlation analysis

The time-lagged correlation between the BSI for each keyword and the daily number of COVID-19 positive tests in China are provided in Supplementary Table S2. Among these keywords, 20 keywords had a maximum lagged correlation coefficient above 0.9, while another 10 keywords had a maximum lagged correlation coefficient ranging from 0.8 to 0.9. A high lagged correlation between the BSI for each keyword and the number of COVID-19 positive tests (See Supplementary Table S2). Therefore, we screened these keywords with lagged correlation coefficients above 0.9 and lag days within 10 days (Table 2), which included 20 keywords in four major categories for subsequent studies to construct predictive models. The correlation was greatest when the BSI of the keyword was 1–10 days earlier than the COVID-19 positive test. Specifically, the keyword “Rhinobyon” reached its maximum lagged correlation at a 1-day lag with a maximum lag correlation coefficient of $\:r\:=\:0.986$ The lagged correlation coefficients for each keyword were statistically tested and were statistically significant.

**Table 2:** Time-lagged correlation analysis between daily number of COVID-19 positive tests and BSI for different keywords

Keyword (in Chinese)	Maximum Correlation coefficient ($\:r)$^a^	Lag days^b^
Respiratory Symptoms (*n* = 6)		
Cough	0.975	0
Dry cough	0.970	1
Expectoration	0.922	0
Rhinobyon	0.986	1
Pharyngalgia	0.945	5
Sore throat	0.973	2
Systemic Symptoms (*n* = 7)		
Fever (Fa Shao)	0.972	4
General malaise	0.915	2
Fatigue (Fa Li)	0.907	2
Myalgias	0.962	5
Headache (Tou Tong)	0.976	3
Headache (Tou Teng)	0.961	3
Dizziness	0.924	1
Other Symptoms (*n* = 2)		
Rash	0.903	1
Taste	0.972	0
Others (*n* = 5)		
Lianhuaqingwen	0.942	10
Lianhuaqingwen capsule	0.948	8
Ibuprofen	0.979	5
COVID-19 antigen	0.970	6
Mask	0.942	7

^a^*P* values of correlation coefficient for keywords were less than 0.05

^b^The number of lag days when the BSI of each keyword and the number of COVID-19 positive tests reached the maximal correlation

**Fig. 4:**
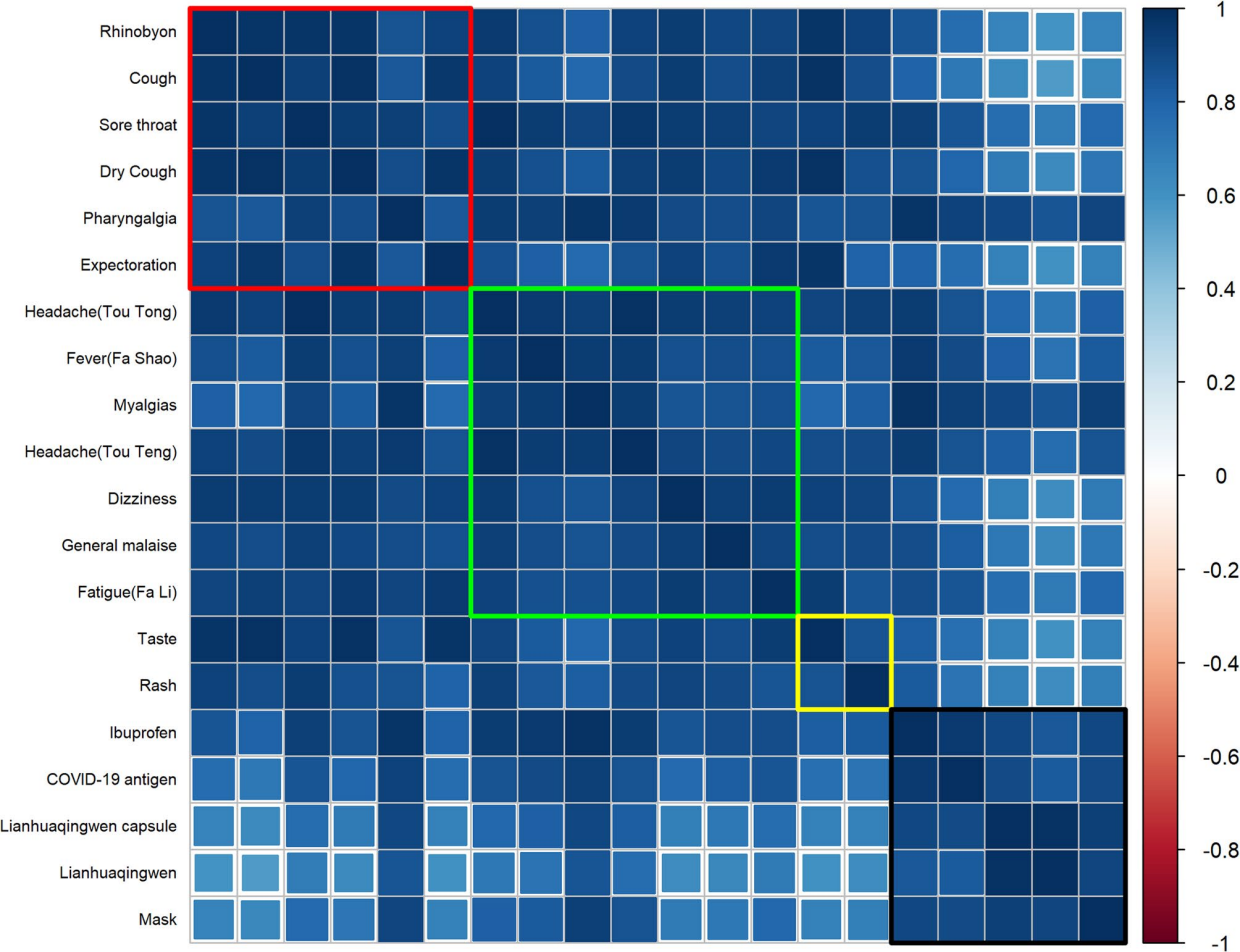
The correlation between the BSI for these included keywords

In addition, we performed Pearson correlation test for each keyword, and its correlation heatmap can be seen in See Supplementary Table S2. We included 20 keywords through time-lagged correlation analysis. Figure 4 shows the correlation between the BSI of these included keywords. Our result showed that there was a significant positive correlation within the same categories of keywords and between different categories of keywords.

The horizontal and vertical labels of the heatmap are 20 keywords; (1) Red box is keywords related to respiratory symptoms; (2) Green box is keywords related to systemic symptoms; (3) Yellow box is keywords related to other symptoms; (4) Black box is keywords related to others; The correlations within each category of keywords are high and statistically significant.

### Predictive models

Table 3 compares the predictive performances of the XGBoost model and the RF model. We used the original BSI dataset and lagged BSI dataset to construct the XGBoost model and RF model to predict the number of COVID-19 positive tests in the future 3, 5, and 7 days, respectively. By comparing the model performance metrics, we found that the RMSE and MAPE values of each XGBoost model were significantly lower than those of the RF model for the same time periods. The predictive capability of XGBoost model was better than that of Random Forest model. In addition, the machine learning models outperformed traditional baselines (ARIMA/ARIMAX; see Supplementary Figure S3), confirming their advantage in short-term prediction accuracy. We found that models constructed using lagged BSI significantly outperformed those using original BSI for predicting over 3, 5, and 7 days. In summary, the XGBoost model showed more accurate predictions compared to the RF model. The optimal model was the XGBoost model constructed using lagged BSI data for forecasting 3 days, with an RMSE value of 803.85 and an MAPE value of 9.96%.

**Table 3:** Comparison of RMSE and MAPE results between XGBoost and random forest models

Periods	XGBoost		Random Forest	
	RMSE	MAPE (%)	RMSE	MAPE (%)
Original BSI ^a^				
3 days	689.89	10.49	2364.76	32.70
5 days	3526.13	79.44	11284.60	147.00
7 days	4488.39	93.21	10473.44	203.99
Lagged BSI ^b^				
3 days	803.85	9.96	2823.34	41.92
5 days	1191.65	18.56	2965.88	50.51
7 days	1832.31	39.76	5279.24	130.55

^a^Original BSI: The original BSI for each keyword as model input

^b^Lagged BSI: The original BSI for each keyword is lagged and then used as input data for the model

**Fig. 5:**
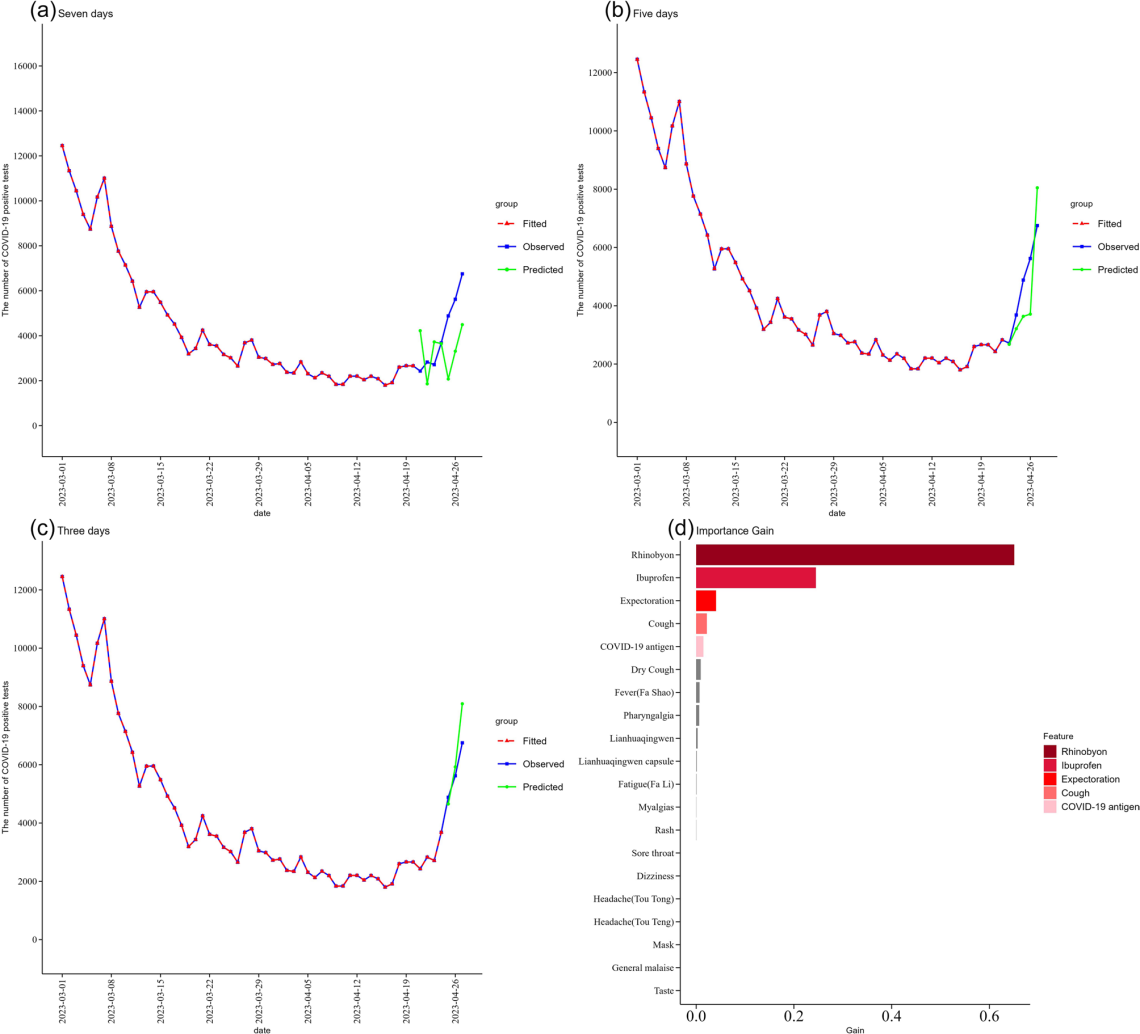
XGBoost model Forecast results and the importance gain of variables **a** 3-day forecast using lagged data; **b** 5-day forecast using lagged data; **c** 3-day forecast using lagged data; **d** Variables importance gain Chart

Figure 5 shows the XGBoost model constructed using the lagged BSI dataset to predict the number of COVID-19 positive tests for the next 3, 5, and 7 days. XGBoost model has the best performance in the 3-day prediction (Fig. 5(c), with predicted values of 4,655 positive tests on April 25, 2023, 5,925 positive tests on April 26, 2023, and 8,092 positive tests on April 27, 2023, respectively. Then, we used this model for feature selection, where 12 variables had importance gain greater than that of the random sequence. We screened 5 keywords that were most important in forecasting the number of COVID-19 positive tests based on their importance gain, namely “Rhinobyon”, “Ibuprofen”, “Expectoration”, “Cough”, “COVID − 19 antigen” (Fig. 5(d). A sensitivity analysis comparing models with the top 5, 7, and 10 features indicated that including additional features did not improve accuracy, confirming the robustness of the 5-feature model (see Supplementary Figure S5, Table S4). To more intuitively illustrate the relationship between online search data and COVID-19 trends, thereby enhancing monitoring and early warning effectiveness, we then weighted the BSI of these keywords according to their importance gain, summed them up, and then normalized the result to finally get a CSI.

### Exposure-response relationships for comprehensive search index with different lag days

**Fig. 6:**
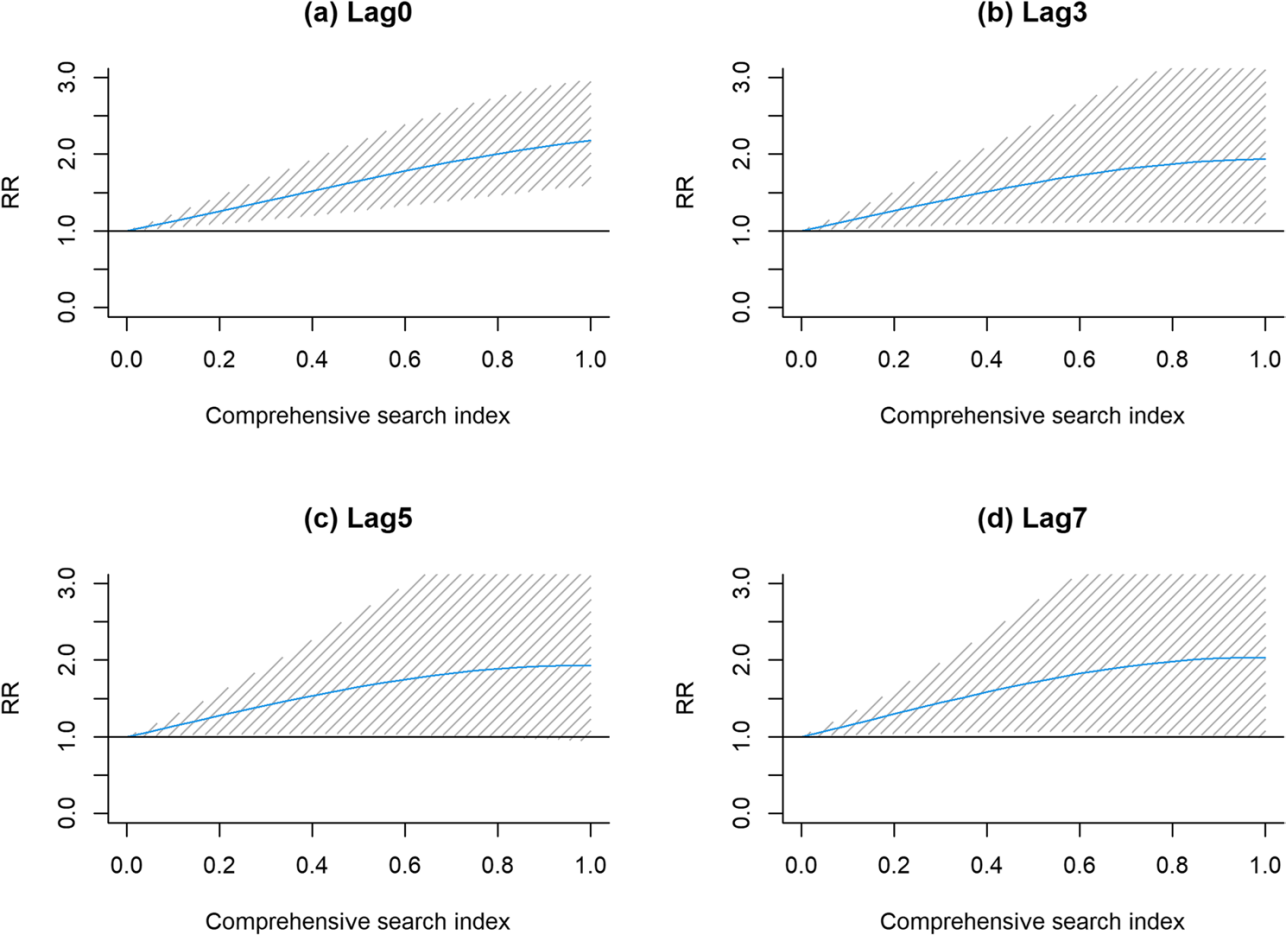
The lag-specific effects of the CSI on the number of COVID-19 positive tests **a**, **b**, **c** and **d** represent the impact of comprehensive search index in the DLNM model on the number of COVID-19 positive tests at lags 0, 3, 5, and 7 days, respectively

The exposure-response effect of the CSI values at lags 0, 3, 5 and 7 days has been shown in the Fig. 6. Overall, the value of the CSI had a statistically significant impact on the number of COVID-19 positive tests. As the CSI increased, the RR at different lag days showed a smooth increasing trend. Specifically, the maximum relative risk (RR) values of the CSI for the number of positive tests at lags of 0, 3, 5 and 7 days were 2.18 (95%*CI* 1.60–2.97), 1.94 (95%*CI* 1.10–3.43), 1.86 (95%*CI* 1.01–3.44) and 2.03 (95%*CI* 1.00–4.11.00.11), respectively.

## Discussion

In this study, we explored the relationship between the BSI for COVID-19-related keywords and the daily number of COVID-19 positive tests in China. We found a consistent time pattern between the BSI for different categories of keywords and the number of COVID-19 positive tests. We also found a high lagged correlation between the BSI and the number of COVID-19 positive tests (*r* > 0.9), with a lag day of 1–10 days, which helped to identify outbreak peaks in advance. In addition, the XGBoost model constructed using lagged BSI enabled relatively accurate predictions of the number of COVID-19 positive tests within the next 3–7 days. We also used feature selection function of XGBoost model to identify the potential keywords that were most relevant in forecasting the number of positive tests during the study period: “Rhinobyon”, “Ibuprofen”, “Expectoration”, “Cough”, “COVID − 19 antigen”. Ultimately, we verified the positive effect of the CSI on the number of positive tests based on the DLNM model, thus providing targeted evidence for decision-making in the real world.

As a new data source for disease surveillance, Internet search data has significant advantages and addresses the shortcomings of traditional surveillance data. Internet search data has become a valuable source for researching population health trends [34]. Traditional surveillance data have a time lag in reporting outbreaks, making it difficult for us to detect an infectious disease outbreak or epidemic in advance [35, 36]. In recent years, the role of Internet search data in early disease surveillance and warning has been proposed. Cuilian et al. [37] found that Google, Baidu, and Sina Weibo could predict COVID-19 outbreaks 1–2 weeks earlier than traditional monitoring systems. Similar results can be observed using other search engine data such as Google Trends [38, 39]. Our study found that BSI for certain keywords peaked 1–44 days earlier than the peak date of the number of COVID-19 positive tests. Our study suggested that the BSI had the ability to identify outbreak peaks at an early stage. This ability may be closely related to the public’s self-diagnosis or information-seeking behavior via the Internet [40]. With China abandoning the clearance policy, public attention to COVID-19 has focused on symptoms and related medications [41]. As observed, the BSI for keywords such as “Fever (Fa Re)”, “Cough”, “Ibuprofen” and others had significant search peaks among various keywords. Interestingly, the BSI for some keywords such as “Diarrhea (Fu Xie)”, “Abdominal pain (Du Zi Teng)”, “Bronchitis”, etc. peaked after the peak data of the number of COVID-19 positive tests, or there was another search peak. In contrast, there were also some keywords for which the BSI fluctuated little. Various factors, including the population search habits, media influence, and public knowledge about COVID-19 [42], might contribute to the diverse temporal patterns among keywords.

There exists a significant lagged correlation between the BSI and the COVID-19 outbreak. Previous studies have identified a correlation between the Internet search data and COVID-19. For example, one study discovered important links between the search terms “COVID”, “COVID pneumonia” and “COVID heart” on Google Trends and daily mortality and morbidity of COVID-19 in the United States [43], with delays of 12 and 19 days, respectively; It also indicated that Google Trends and Baidu search data using COVID-19-related symptom terms were able to forecast daily new confirmed COVID-19 cases over a 12-day period [44]. In this study, we found that the BSI of different keywords had different lagged effect on the number of COVID-19 positive tests. Moreover, a significant correlation was observed between the keywords “Rhinobyon”, “Cough”, “Fever (Fa Shao)”, and “Ibuprofen” and the number of COVID-19 positive tests, with a lag day ranging from 2 to 7 days. These findings consistent with previous research [45]. However, they found that the most relevant keywords were “cough,” “fatigue,” “sputum production,” “shortness of breath,” and “fever,” with different optimal lag times compared to our findings. This suggests that the lag effects of keywords on epidemic trends may vary across different phases of the pandemic, highlighting the need to develop models that account for these shifting dynamics. We hypothesized that this lagged effect might result from the fact that the Chinese COVID-19 patients within our study were predominantly mildly ill, and that most of these symptoms manifested as rhinobyon, cough, and fever [46]. Furthermore, none of the collected keywords related to gastrointestinal symptoms were correlated with the daily number of COVID-19 positive tests, indicating that Chinese residents were unlikely to experience gastrointestinal symptoms due to COVID-19 infection. Previous studies have also shown that gastrointestinal symptoms occur in only a minority of patients with COVID-19 infections and vary across study populations [47]. When we compared this with studies from other time periods, we found that during various phases of the COVID-19 pandemic, changes in public attention to COVID-19 might modify the association between COVID-19 and Internet search data. Given that our study was conducted during a relatively stable post-policy-relaxation period, when China had already fully lifted nationwide COVID-19 control measures, the observed alignment and lagged correlation between BSI and cases likely reflect underlying epidemic dynamics rather than short-term media or policy shocks. Nonetheless, we also acknowledge that certain search behaviors may be driven more by public anxiety than actual infections, which may introduce which may introduce certain confounding factors, such as some irrelevant hotwords. Therefore, it is essential to implement real-time updates and optimization of keyword selection strategies to ensure the timeliness and relevance of the findings. Overall, in future research we need to focus on the dynamics of Internet search data in different epidemic contexts, and explore how to use these data more accurately to optimize epidemic surveillance and prediction models.

Using Internet search data to construct machine learning predictive models can serve as a robust complement to traditional monitoring systems, helping us to identify potential predictors in advance. Traditional predictive models are mostly based on clinical or virologic data [48, 49], which are often time-consuming, costly, and inefficient. In contrast, utilizing Internet search data can significantly reduce the time and resources required for data collection. In recent years, researchers have increasingly employed Internet search data to construct forecasting models. Google Trends has been used by countries for many years to estimate influenza-like illnesses to supplement traditional surveillance systems [50, 51]. During the COVID-19 pandemic, studies have also been conducted to construct a COVID-19 pandemic surveillance system based on social media data to accurately and proactively forecast pandemic trends [52]. A literature review also found that using Google Trends significantly enhanced the predictive power of several analysis methods [53]. Baidu Index was also successfully utilized for timely and accurate forecasting of COVID-19 [54, 55]. In our study, we evaluated the effectiveness of two machine learning models in predicting the future number of COVID-19 positive tests. The results showed that the XGBoost model outperformed the RF model. The prediction models developed using the lagged BSI had better prediction performance than using the original BSI. The XGBoost model performed best in the short-term predictions of 3 days (Fig. 5(c)) and was able to make more accurate quantitative predictions. The lagged BSI could capture the trend of epidemic development more accurately. Short-term forecasting (e.g., 3–7 days) has been widely adopted in COVID-19 surveillance [56, 57], as they provide timely and actionable information. While longer-term forecasts (e.g., 14 or 30 days) were also tested (see Supplementary Figure S4), performance declined modestly yet still outperformed most alternative models [58]. This suggests that reduced coupling between search behavior and future case trends, along with limited training data coverage and the inherent uncertainty of infectious disease dynamics, may compromise the accuracy of long-term forecasts. Future improvements could involve incorporating mobility, behavioral, or intervention data to enhance long-term forecasting capability. Our finding suggested that in similar epidemic scenarios, employing the XGBoost model combined with lagged BSI might be a more effective approach, enhancing predictive accuracy and timeliness.

In addition, the feature selection function of the XGBoost model identified five keywords: “Rhinobyon”、“Ibuprofen”、“Expectoration”、“Cough”、“COVID-19 antigen”. Among these predictors, “Rhinobyon”、“Cough”、“Expectoration” were relatively common symptoms during the study period, while “Ibuprofen”、“COVID-19 antigen” reflected the behavior of individuals who started to actively seek medical care after the liberalization of prevention and control policies. Interestingly, some common symptom keywords, such as “Fever (Fa Shao)” and “Headache (Tou Teng)” had little impact on the predictive performance of the model. This suggested that not all Internet search index for keywords could be used as predictors. People may use different names or terms to describe the same symptom or sign in their daily lives. Additionally, an increase in searches may arise due to similar symptoms of other diseases at certain times. It is yet to be tested whether the selected keywords can be used to predict COVID-19.

Previous studies have found that there are many factors that can affect the trend of the epidemic [59, 60] making a single keyword insufficient for effective decision-making. Considering the relative importance of predictive factors, we constructed a CSI by integrating and weighting multiple variables. It was found that an increase in the CSI at different lags led to a nonlinear increase in the number of positive tests. As the lag period increased, the effect of the CSI diminished over time. This lagged effect of the CSI indicates that the keywords we identified through the machine learning model can be used as effective predictors to realize the early monitoring and alerting of COVID-19. By focusing on one or a few key keywords, CSI can more effectively reflect the trend of the epidemic. This method not only facilitates the implementation of public health decisions, but also enhances the efficiency and responsiveness of monitoring systems, providing a scientific basis for the allocation of public health resources and the optimization of preventive measures.

### Strengths and limitations

Based on the above research, our study has four major strengths. First, we were the first to verify the ability of the BSI to provide early warning of COVID-19 in China after active screening for COVID-19 was eliminated. Second, we include as many relevant keywords as possible and screened out potential predictors. Third, we demonstrate that the XGBoost model using lagged BSI is more effective for predicting COVID-19 epidemic. Fourth, our CSI provides an intuitive reflection of epidemic trends, thereby aiding public health decision-making. However, several limitations in our research must be acknowledged. First, lower internet usage among older adults and in rural areas may introduce bias by underrepresenting the behaviors of these groups. While family members or caregivers may be able to conduct searches on their behalf, thereby reducing bias across populations to some extent, future research must delve more deeply into the search behaviors of different social groups to gain a more comprehensive understanding of these effects. In addition, the model was not externally validated, which limits its generalizability. Future research should address this by conducting validation using datasets from different time periods or geographic regions.

## Conclusions

In conclusion, our results showed that after the elimination of active surveillance for COVID-19, the XGBoost model using lagged BSI could better predict the COVID-19 epidemics and identify potential predictors. Internet search data have proven crucial role in the early surveillance and timely warning of emerging infectious diseases. Our findings have important implications for predicting and responding to similar outbreaks in the future. When an emerging infectious disease occurs next, proactive monitoring of the BSI for relevant keywords in advance and fitting the XGBoost predictive model can assist health departments in conducting rapid and accurate surveillance, early warning, and strategic health resource allocation before disease outbreaks.

## Supplementary Information

Supplementary Material 1

## Data Availability

The datasets analysed during the current study are available in the website as follows: (https://www.chinacdc.cn/jksj/xgbdyq/202411/t20241112_302588.html) and (https://index.baidu.com).

## Abbreviations

BSI: Baidu search index
CCF: cross-correlation function
XGBoost: extreme gradient boost
RF: random forest
RMSE: root mean squared error
MAPE: mean absolute percentage error
CSI: comprehensive search index
DLNM: distributed lagged nonlinear model
