# Supplementary material for: Developing predictive models for COVID-19 positive tests based on the XGBoost and random forest algorithms with internet search data
Source: BMC Public Health. 2025 Nov 28;25:4189. doi: 10.1186/s12889-025-25569-w (PMC12664191; doi:10.1186/s12889-025-25569-w)
Supplement: Supplementary file 1 — Supplementary Material 1 [file 12889_2025_25569_MOESM1_ESM.docx]

**Supplementary materials**

**Supplementary Table S1** Catalog of keywords included in the study

| **Category** | **Keyword (in Chinese)**^a^ |
| --- | --- |
| **COVID-19 terms (n=4)** |  |
|  | Novel Coronavirus Pneumonia |
|  | Novel Coronavirus Infection |
|  | COVID-19 (Xin Guan Fei Yan) |
|  | COVID-19 (Xin Guan Gan Ran) |
| **Respiratory Symptoms (n=18)** |  |
|  | Cough |
|  | Dry cough |
|  | Expectoration |
|  | Rhinorrhea (Liu Ti) |
|  | Rhinorrhea (Liu Bi Ti) |
|  | Rhinobyon |
|  | Sneeze (Da Pen Ti) |
|  | Sneeze (Pen Ti) |
|  | Pharyngalgia |
|  | Hoarseness |
|  | Acute laryngitis |
|  | Bronchitis |
|  | Wheeze |
|  | Asthma |
|  | Sore throat |
|  | Hypopnea |
|  | Dyspnea |
|  | Shortness of breath |
| **Systemic Symptoms (n=18)** |  |
|  | Chest tightness |
|  | Chest pain |
|  | Palpitate (Xin Huang) |
|  | Palpitate (Xin Ji) |
|  | Fever (Fa Re) |
|  | Fever (Fa Shao) |
|  | Rigor (Han Chan) |
|  | Rigor (Han Zhan) |
|  | General malaise |
|  | Fatigue (Fa Li) |
|  | Fatigue (Pi Lao) |
|  | Myalgias |
|  | Headache (Tou Tong) |
|  | Arthralgia |
|  | Backache |
|  | Headache (Tou Teng) |
|  | Dizziness |
|  | Vertigo |
| **Gastrointestinal Symptoms (n=7)** |  |
|  | Abdominal pain (Fu Tong) |
|  | Abdominal pain (Du Zi Tong) |
|  | Abdominal pain (Du Zi Teng) |
|  | Diarrhea (Fu Xie) |
|  | Diarrhea (La Du Zi) |
|  | Nausea |
|  | Vomiting |
| **Other Symptoms (n=11)** |  |
|  | Rash |
|  | Conjunctivitis |
|  | Eyeball congestion |
|  | Conjunctival congestion |
|  | Red eyes |
|  | Itchy eyes |
|  | Eyes hurt (Yan Jing Teng) |
|  | Eyes hurt (Yan Jing Tong) |
|  | Smell |
|  | Taste |
|  | Epilepsy |
| **Others (n=9)** |  |
|  | Lianhuaqingwen |
|  | Lianhuaqingwen capsule |
|  | Isolation |
|  | COVID-19 vaccine |
|  | Ibuprofen |
|  | Nasopharyngeal swab |
|  | COVID-19 antigen |
|  | Nucleic Acids Detection |
|  | Mask |

^a^When different keywords are the same in English, the keywords are displayed in English (Chinese pinyin) format.

**Supplementary Table S2** Correlation Analysis Between the Daily Number of COVID-19 Positive Tests and Baidu Search Index for Initial Keywords

| **Keyword (in Chinese)** | **Maximum Correlation coefficient (**$r$**)^a^** | **Lag days^b^** |
| --- | --- | --- |
| **COVID-19 terms** |  |  |
| Novel Coronavirus Pneumonia | 0.209 | 10 |
| Novel Coronavirus Infection | 0.424 | 0 |
| COVID-19 (Xin Guan Fei Yan) | 0.801 | 4 |
| COVID-19 (Xin Guan Gan Ran) | 0.387 | 0 |
| **Respiratory Symptoms** |  |  |
| Cough | 0.975 | 0 |
| Dry cough | 0.970 | 1 |
| Expectoration | 0.922 | 0 |
| Rhinorrhea (Liu Ti) | 0.771 | 2 |
| Rhinorrhea (Liu Bi Ti) | 0.858 | 3 |
| Rhinobyon | 0.986 | 1 |
| Sneeze (Da Pen Ti) | 0.816 | 0 |
| Sneeze (Pen Ti) | 0.397 | 4 |
| Pharyngalgia | 0.945 | 5 |
| Hoarseness | 0.612 | 1 |
| Acute laryngitis | 0.827 | 0 |
| Bronchitis | 0.678 | 0 |
| Wheeze | 0.362 | 0 |
| Asthma | 0.817 | 0 |
| Sore throat | 0.973 | 2 |
| Hypopnea | 0.717 | 0 |
| Dyspnea | 0.811 | 0 |
| Shortness of breath | 0.700 | 0 |
| **Systemic Symptoms** |  |  |
| Chest tightness | 0.682 | 0 |
| Chest pain | 0.410 | 0 |
| Palpitate (Xin Huang) | 0.513 | 0 |
| Palpitate (Xin Ji) | 0.479 | 0 |
| Fever (Fa Re) | 0.795 | 6 |
| Fever (Fa Shao) | 0.971 | 4 |
| Rigor (Han Chan) | 0.889 | 2 |
| Rigor (Han Zhan) | 0.202 | 1 |
| General malaise | 0.915 | 2 |
| Fatigue (Fa Li) | 0.907 | 2 |
| Fatigue (Pi Lao) | 0.280 | 2 |
| Myalgias | 0.962 | 5 |
| Headache (Tou Tong) | 0.976 | 3 |
| Arthralgia | 0.804 | 4 |
| Backache | 0.383 | 5 |
| Headache (Tou Teng) | 0.961 | 3 |
| Dizziness | 0.924 | 1 |
| Vertigo | 0.087 | 0 |
| **Gastrointestinal Symptoms** |  |  |
| Abdominal pain (Fu Tong) | 0.005 | 10 |
| Abdominal pain (Du Zi Tong) | 0.141 | 6 |
| Abdominal pain (Du Zi Teng) | 0.105 | 2 |
| Diarrhea (Fu Xie) | 0.580 | 3 |
| Diarrhea (La Du Zi) | 0.368 | 1 |
| Nausea | 0.079 | 1 |
| Vomiting | 0.744 | 1 |
| **Other Symptoms** |  |  |
| Rash | 0.903 | 1 |
| Conjunctivitis | 0.399 | 8 |
| Eyeball congestion | 0.163 | 10 |
| Conjunctival congestion | 0.116 | 10 |
| Red eyes | 0.650 | 5 |
| Itchy eyes | -0.050 | 10 |
| Eyes hurt (Yan Jing Teng) | 0.723 | 3 |
| Eyes hurt (Yan Jing Tong) | 0.881 | 3 |
| Smell | 0.852 | 0 |
| Taste | 0.972 | 0 |
| Epilepsy | -0.147 | 10 |
| **Others** |  |  |
| Lianhuaqingwen | 0.942 | 10 |
| Lianhuaqingwen capsule | 0.948 | 8 |
| Isolation | 0.314 | 10 |
| COVID-19 vaccine | 0.608 | 10 |
| Ibuprofen | 0.979 | 5 |
| Nasopharyngeal swab | 0.583 | 10 |
| COVID-19 antigen | 0.970 | 6 |
| Nucleic Acids Detection | 0.688 | 10 |
| Mask | 0.942 | 7 |

^a^The number of lag days when the BSI of each keyword and the number of COVID-19 positive tests reached the maximal correlation.

^b^The number of lag days when the BSI of each keyword and the number of COVID-19 positive tests reached the maximal correlation.


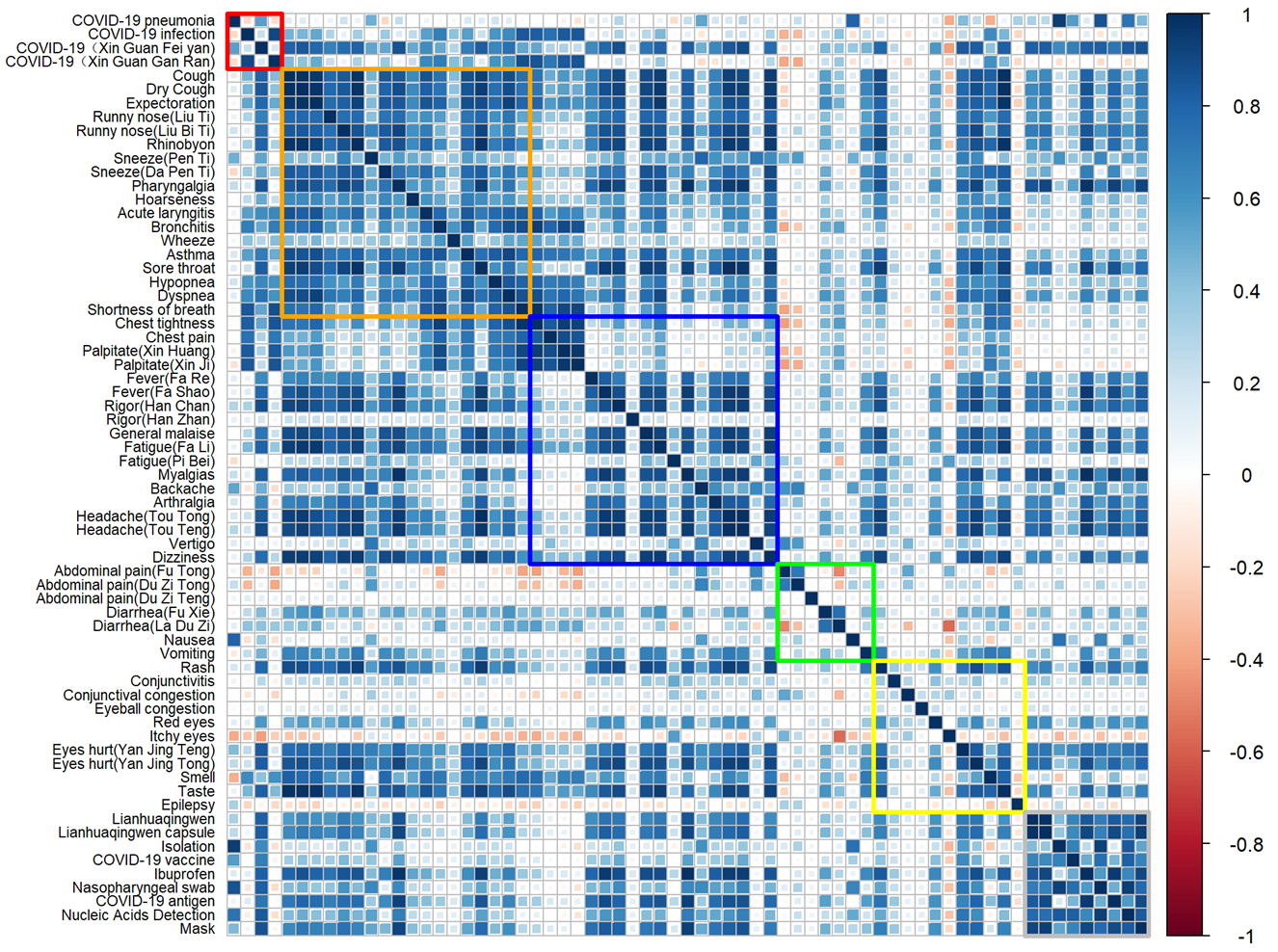


**Supplementary Figure S1** The Correlation Among Initial Keywords

The horizontal and vertical labels of the heatmap are 67 keywords; (1) Red box is keywords related to COVID-19 terms; (2) Orange box is keywords related to respiratory symptoms; (1) Red box is keywords related to COVID-19 terms; (3) Blue box is keywords related to systemic symptoms; (4) Green box is keywords related to gastrointestinal symptoms; (5) Yellow box is keywords related to other symptoms; (6) Grey box is keywords related to others.

**Supplementary Figure S2 XGBoost model Forecast results**

(a) 3-day forecast using CSI; (b) 5-day forecast using CSI; (c) 3-day forecast using CSI;

**Supplementary Table S3 Comparison of RMSE and MAPE results between constructing XGBoost model with a single indicator or XGBoost model with CSI**

| **Periods** | **BSI** | | **CSI** | |
| --- | --- | --- | --- | --- |
|  | **RMSE** | **MAPE (%)** | **RMSE** | **MAPE (%)** |
| 3 days | 803.85 | 9.96 | 4094.28 | 71.71 |
| 5 days | 1191.65 | 18.56 | 3181.12 | 49.38 |
| 7 days | 1832.31 | 39.76 | 8690.73 | 227.45 |


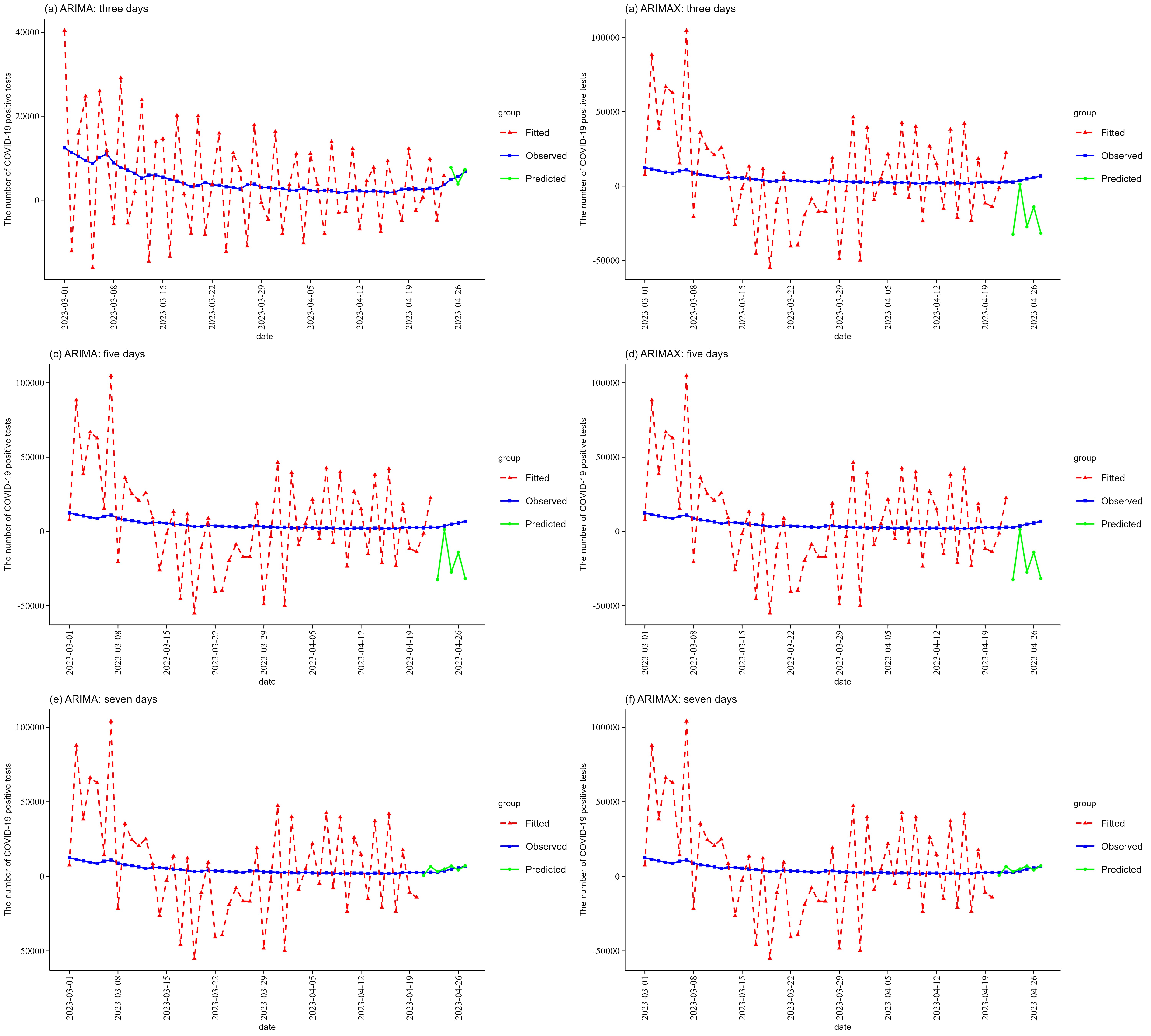


**Supplementary Figure S3 Prediction results using the ARIMA/ARIMAX model with lagged Baidu Search Index**

We constructed traditional time series models such as ARIMA and ARIMAX models for 3, 5, and 7 forecasts. The figures are as follows: (a) ARIMA model forecast for 3 days; (b) ARIMA model forecast for 3 days; (c) ARIMA model forecast for 5 days; (d) ARIMAX model forecast for 5 days; (e) ARIMA model forecast for 7 days; (f) ARIMA model forecast for 7 days.


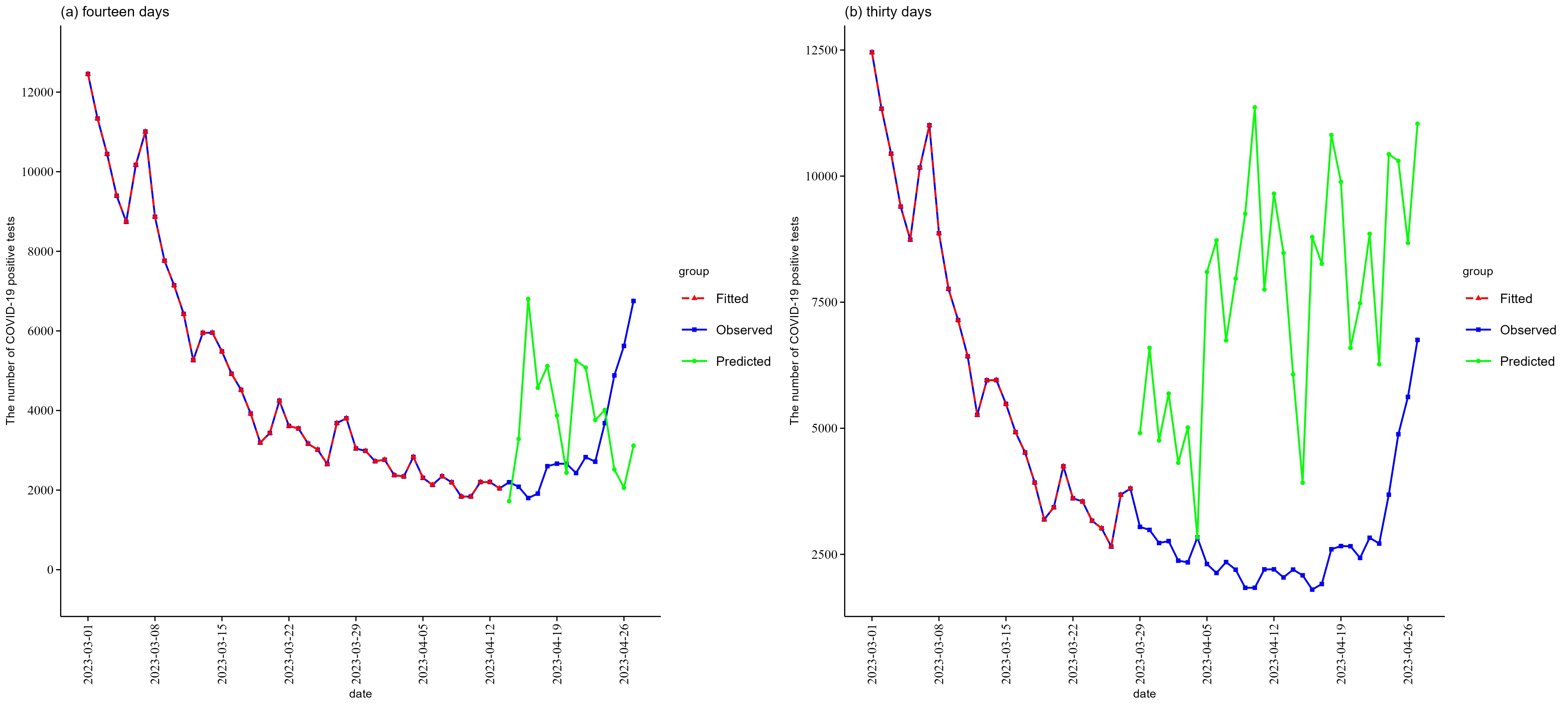


**Supplementary Figure S4 XGBoost model Forecast results**

1. 14-day forecast using lagged data, RMSE: 2497.47, MAPE: 75.4%; (b) 30-day forecast using lagged data, RMSE: 5363.02, MAPE: 202.6%.

**Supplementary Figure S5 Performance of the Reconstructed XGBoost Model with Selected Features for Predicting COVID-19 Positivity Rates**

1. 3-day forecast using selected 5 features, RMSE: 1130.5, MAPE: 16.0%; (b) 5-day forecast using selected 5 features, RMSE: 1214.2, MAPE: 25.2%; (c) 7-day forecast using selected 5 features, RMSE: 1727.4, MAPE: 38.2%; (d)3-day forecast using selected 10 features, RMSE: 1184.4, MAPE: 20.9%; (e) 5-day forecast using selected 10 features, RMSE: 1264.1, MAPE: 26.3%; (f) 7-day forecast using selected 10 features, RMSE: 2420.2, MAPE: 47.3%; (g) 3-day forecast using selected 7 features, RMSE: 1770.7, MAPE: 25.1%; (h) 5-day forecast using selected 7 features, RMSE: 1820, MAPE: 31.8%; (i) 7-day forecast using selected 7 features, RMSE: 2701.6, MAPE: 48.8%.

**Supplementary Table S4 Comparison of XGBoost Prediction Performance Using Different Features**

|  | Five Features | | Seven Features | | Ten Features | |
| --- | --- | --- | --- | --- | --- | --- |
|  | RMSE | MAPE(%) | RMSE | MAPE(%) | RMSE | MAPE(%) |
| Three days | 1130.5 | 16.0 | 1770.7 | 25.1 | 1184.4 | 20.9 |
| Five days | 1214.2 | 25.2 | 1820 | 31.8 | 1264.1 | 26.3 |
| Seven days | 1727.4 | 38.2 | 2701.6 | 48.8 | 2420.2 | 47.3 |
